# Relationships among children's muscular strength, neuromuscular control, and resilience

**DOI:** 10.3389/fped.2026.1743647

**Published:** 2026-03-06

**Authors:** Lauren M. Wagner, Phil Esposito, Robyn Braun-Trocchio, Hailey G. von Borck, Deborah J. Rhea

**Affiliations:** Department of Kinesiology, Texas Christian University, Fort Worth, TX, United States

**Keywords:** assessment, child performance, fitness, muscular strength, neuromuscular control, resilience

## Abstract

**Introduction:**

Children who participate in recess interventions demonstrate increased upper- and lower-body muscular strength (MusS), neuromuscular control (NC), and resilience. However, to measure the effectiveness of recess interventions, identifying the relationship between physical and psychological assessments is necessary for reliability and consistency. Therefore, this study explores the relationships and variable predictions among MusS, NC, and resilience assessments in fourth- and fifth-grade children at two time points during one school year.

**Methods:**

A total of 164 fourth- and fifth-grade children participated in MusS (standing broad jump, push-ups, single-leg three-hop, and average grip strength), NC (side-step), and resilience (Child and Youth Resilience Measure-Revised) assessments. A single intraclass correlation coefficient was calculated in September (Time 1) and January (Time 2) to evaluate the reliability of each assessment. Next, Pearson product correlations and multiple linear regression analyses were conducted to assess outcomes.

**Results:**

Times 1 and 2 had positive, moderate, and significant correlations among the following MusS assessments: standing broad jump and average single-leg three-hop, standing broad jump and push-up, and average single-leg three-hop and push-up (*p* < 0.01). NC and resilience had no meaningful correlations with the other assessments. Regression analyses further revealed that push-up performance was a significant predictor of standing broad jump performance at Times 1 and 2 (*p* < 0.001).

**Conclusions:**

MusS assessments were practical, reliable, time-efficient, and low cost for this age group. Therefore, these elements should be taken into consideration to measure the effectiveness of recess or physical activity interventions. In addition, the results revealed correlations between upper- and lower-body MusS assessments. The relationship between the standing broad jump and push-ups highlights the interconnective nature of the upper and lower MusS. This raises the question of why resilience and NC were not related to each other or to MusS assessments. Given the exploratory nature and short duration of this study, further research is needed to validate these findings and to determine whether there are more effective NC and resilience assessments to use with this age group.

## 1 Introduction

Childhood is a fundamental period for achieving physical development through motor skills, muscular strength (MusS), neuromuscular control (NC), and overall movement competency (1–3). When provided with self-directed outdoor opportunities to engage in physical activity (PA), children develop a broader sense of wellbeing and social engagement, along with movement skills such as running, climbing, and jumping, which serve as building blocks for advanced movements later in life (2, 4, 5). Although many PA interventions target singular sports and structured physical education (PE), recess interventions that offer unstructured outdoor play remain viable alternatives (2, 3, 6–11). Specifically, when conducted in an equitable and safe environment, recess interventions can promote physical and psychological improvements (2, 3, 6–11).

Theorists such as Jean Piaget and Peter Gray have emphasized play as a major environmental contributor to the holistic development of the child (5, 12–14). Play comes very naturally to children when accessible, as seen during recess; however, the physical and psychological benefits vary based on sex and age. According to Piaget's Cognitive Development Theory, individuals move through four developmental stages from early childhood to adulthood (13). The concrete operational stage, which focuses on 7- to 11-year-old children, is characterized by the development of basic reasoning as well as logic and increased physical activity (PA) (13, 15, 16). PA includes unilateral, bilateral, and contralateral movements, which have been shown to build MusS and promote higher bone density, resulting in a decrease in chronic disease, fatigue, and risk of injury (11, 17–21). This study focuses on this age range, specifically 9–11 (fourth and fifth graders), which is unique as it precludes maturation; thus, children appear to be more similar than different, as sex-specific factors such as height, weight, flexibility, and muscle mass are less pronounced (22, 23). Therefore, the concrete operational stage is a period of reduced bias for understanding children's physical and psychological development prior to maturation.

In addition, NC is essential for physical wellbeing and is observed in children when they engage in recess, defined as unstructured outdoor play (11, 21). NC is the brain–body connection essential for healthy physical development, seen in unstructured outdoor play, through coordination, balance, and agility (2, 3, 21). NC is fundamental in environments such as school recess, where fast-paced movements occur, serving as the foundation for more advanced settings, including PA, fitness lifestyles, and sports participation. In the absence of NC, there is an increased risk of falls and injuries (2). In recent child literature, the impact of NC driven by sex and age remains unclear. Webb et al. (2) and Wagner et al. (21) found no sex differences for NC. However, these studies remain inconclusive on whether there are NC differences by age.

Beyond physical benefits, recess may also contribute to the psychological development of children, specifically resilience, which is the ability to cope and recover in the face of adversity, stress, and challenges (24–26). Physical challenges experienced during recess are meaningful opportunities for children to encourage the development of resilience, build confidence, and improve emotion regulation (27). In addition, recess provides peer interactions that promote resilience in self-directed and natural environments (28). For 9- to 11-year-olds, resilience appears to remain stable across sex (21). However, further research is needed to fully understand resilience in this population by age and sex.

Despite the known benefits of recess, few studies have examined the relationships among MusS, NC, and resilience assessments, particularly in the context of evaluating the outcomes of recess interventions. Most existing literature assesses sex, grade, or recess intervention differences within these variables; however, understanding the relationships of the chosen physical and psychological assessments is valuable to further understand child development and help provide more consistency when measuring the effectiveness of recess interventions. Thus, this is an exploratory study to assess any relationships or variable predictions among MusS, NC, and resilience in fourth- and fifth-grade children at two time points during one school year.

## 2 Methods

This study is a secondary quantitative analysis derived from a larger pretest–posttest study to evaluate relationships among MusS, NC, and resilience and/or variable predictions for recess intervention assessment use in the future (21). The University Institutional Review Board approved the study’s ethics, and elementary school administrators approved the school data collection process. School administrators, parents, and children were fully informed of the study's purpose, and informed consent was obtained from parents and assent from children prior to participation. Furthermore, pilot testing was conducted in the spring of 2024, which validated the assessment tool, demonstrated rater inter-rater reliability, and ensured appropriateness in the target population.

### 2.1 Participants

A total of 164 children from three North Texas elementary schools participated in this larger LiiNK Project® pretest–posttest modified recess intervention study (21). Children received 30 min of recess and 15 min of character development lessons daily. The children also participated in PE every other day for 50 min. Data for this study were collected in early September 2024 and late January 2025. Table 1 presents the grade, sex, and race demographics for this study.

**Table 1:** Demographics for grade, sex, and race of the participants.

Grade	Sex	Race		Total
		White	POC	
Fourth	Female	16	27	43
	Male	18	21	39
	Total	34	48	82
Fifth	Female	18	21	39
	Male	15	28	43
	Total	33	49	82
Total	Female	34	48	82
	Male	33	49	82
	Total	67	97	164

POC, person of color.

The inclusion criteria were (1) parental consent and child assent, (2) fourth- and fifth-grade children, who were 9–11 years old, (3) no injuries at the time of participation that prevented them from participating in PE class, (4) the ability to read and speak English, and (5) completion of the physical and psychological assessments at Times 1 and 2. Children were excluded from the study if they did not meet the inclusion criteria, withdrew from participation at any time during the data collection process, and/or did not participate in the modified LiiNK Project® recess intervention.

An *a priori* power analysis was conducted using G*Power 3.1 to determine the minimum sample size required to test the study hypothesis (using *a* = 0.05, *b* = 0.80, and *ƒ* = 0.25). The results were computed for 86 children with adequate statistical power. At Time 1, the original sample size included 182 children, with anticipation of attrition between Times 1 and 2. The final sample consisted of 164 children.

### 2.2 Instrumentation

#### 2.2.1 Dynamometer grip strength

The single-hand grip strength test is an upper-body unilateral test that is associated with the prediction of upper-body strength, musculoskeletal fitness, bone mineral density, malnutrition, diabetes, and quality of life (2, 21, 29).

#### 2.2.2 Push-up

The push-up test is a standard predictor and developer of the arms, shoulders, and chest. It assesses the strength resistance of the elbow flexors and targets the measurement of children's strength endurance (30, 31). This test is a common recurring test to evaluate physical capacity in children, adolescents, and adults, which can be seen throughout fitness batteries such as FitnessGram (2, 21, 32).

#### 2.2.3 Single-leg three hop

The single-leg three-hop test measures power and MusS unilaterally. For research and clinical studies, it is used to assess PA readiness and assist in determining recovery and ability to return to sport safely (2, 21, 33, 34). Lower scores are often associated with an increased risk of thigh, knee, and ankle injury.

#### 2.2.4 Standing broad jump

The standing broad jump is a well-known, reliable, and valid test for assessing explosive bilateral lower-body MusS and physical fitness (35, 36). Explosive strength, also known as muscular power, is predictive of lipid metabolic profiles, cardiovascular health, skeletal health, and adiposity (21, 35–37). It measures the anaerobic power of the lower limbs while monitoring strength gains to determine sports and PA abilities (36).

#### 2.2.5 Side-step

The side-step test assesses the lower-body NC. This test allows for a greater understanding of the brain–body connection through the recruitment of the lower body and core muscles (2, 3, 21, 38). It also examines agility by moving quickly in different directions, while maintaining balance (39).

#### 2.2.6 Child and Youth Resilience Measure-Revised

The Child and Youth Resilience Measure-Revised (CYRM-R) is a valid and reliable Rasch-validated updated measure of the Child and Youth Resilience Measure (40). The CYRM-R consists of 17 items on a five-point Likert scale (21, 40, 41). All items were summed to obtain a total score of the child's resilience, with a minimum score of 17 and a maximum score of 85. For this study, the Cronbach’s alpha for total resilience at both time points was 0.87, which is considered acceptable (40). The children received the fully revised measure at both time points.

### 2.3 Procedure

A research team with three to five members administered the CYRM-R on the provided computers at one station in conjunction with physical assessment stations during the child's designated PE class. The CYRM-R was completed in the gymnasium office to minimize noise and distractions from regular PE activities. The children were provided instructions in one large group and then divided into smaller groups to begin testing at each station. The researchers remained at their assigned stations, while the children rotated through the stations.

### 2.4 Data analysis

The data were cleaned in Microsoft Excel and analyzed using IMB Statistical Package for Social Sciences (SPSS) Version 29. Descriptive statistics (mean ± SD) were calculated for the variables studied. The test–retest reliability between MusS and NC at Times 1 and 2 was calculated using the intraclass correlation coefficient (ICC) based on a two-way mixed-effects model with absolute agreement for single measurements. The ICC was interpreted using conventional cutoffs, with values below 0.50 indicating poor reliability, values between 0.50 and 0.75 indicating moderate reliability, values between 0.75 and 0.90 indicating good reliability, and values above 0.90 indicating excellent reliability (42). Pearson product correlation coefficients were used to examine the relationships among MusS, NC, and resilience for the Times 1 and 2 scores. If moderate to strong and statistically significant correlations were present (*r* > 0.50, *p* < 0.05), it suggested that these variables explained a substantial amount of variance in the data. Thus, a multiple linear regression model was deemed appropriate for assessing the predictors. To assess multicollinearity among the independent variables, highly correlated predictors were removed to simplify the model and reduce the multicollinearity. This approach improved model stability and interpretability while preserving the most meaningful predictors for multicollinearity, which was also examined using variance inflation factors (VIFs), condition indices, eigenvalues, and variance proportions. VIFs above five were considered indicative of potential multicollinearity. In addition, a condition index ranging from 10 to 30 or above 30, particularly when accompanied by high variance proportions (>0.5) for two or more variables on the same dimension, signaled serious multicollinearity. If the variables exhibited these characteristics, further investigation was conducted.

## 3 Results

Singular ICCs were calculated for each MusS, NC, and resilience assessment by combining the Times 1 and 2 data for each assessment. The average grip strength [ICC(2,1) = 0.86, 95% CI (0.84, 0.88)], push-up [ICC(2,1) = 0.78, 95% CI (0.75, 0.81)], single-leg three-hop [ICC(2,1) = 0.81, 95% CI (0.78, 0.83)], and standing broad jump [ICC(2,1) = 0.81, 95% CI (0.78, 0.83)] indicated good reliability, while side-step indicated moderate reliability [ICC(2,1) = 0.53, 95% CI (0.47, 0.59)].

Pearson product correlations among MusS, NC, and resilience scores were examined for the Times 1 and 2 scores. Table 2 presents the means, standard deviations, and Pearson product correlations for the Time 1 variables. Positive, moderate, and statistically significant correlations were found among the following MusS assessments: standing broad jump and average single-leg three-hop [*r* (163) = 0.75, *p* < 0.01]; standing broad jump and push-up [*r* (163) = 0.60, *p* < 0.01]; and average single-leg three-hop and push-up [*r* (163) = 0.59, *p* < 0.01]. Meanwhile, resilience had positive, weak, and non-significant correlations with all MusS assessments and NC. Resilience was positive, weak, and significantly correlated to the side-step test (NC) [*r* (163) = 0.20, *p* = 0.05].

**Table 2:** Relationship among MusS, NC, and resilience at Time 1.

Variable	*M*	SD	1	2	3	4	5
1. Average grip strength	34.96	8.32					
2. Push-up	14.28	10.36	0.15 [−0.01, 0.29]				
3. Average single-leg three-hop	100.33	29.46	0.30** [0.16, 0.43]	0.59** [0.48, 0.68]			
4. Standing broad jump	48.56	10.61	0.37** [0.23, 0.49]	0.60** [0.50, 0.69]	0.75** [0.68, 0.81]		
5. Side-step	26.43	5.76	0.10 [−0.06, 0.25]	0.34** [0.20, 0.47]	0.42** [0.29, 0.54]	0.33** [0.18, 0.46]	
6. Resilience	70.54	10.47	0.11 [−0.04, 0.26]	0.12 [−0.04, 0.26]	0.14 [−0.02, 0.28]	0.14 [−0.01, 0.29]	0.20* [0.05, 0.34]

* *p* < .05.

** *p* < .01.

An inspection of the Time 1 variables revealed that the single-leg three-hop, push-up, and average grip strength were highly correlated to and predictive of the standing broad jump [*R*^2^ = 0.63, adjusted *R*^2^ = 0.62, *F* (1, 162) = 10.48, *p* = 0.001]. Although diagnostic indices suggested that multicollinearity was not severe (variance inflation facto*r* = 0.91, eigenvalue ≤3.69, and condition index = 11.95), the single-leg three-hop was removed from the regression analysis because of the greatest correlation. Meanwhile, the average grip strength was not included in the model to improve model parsimony, since the average grip strength added only 2.2% variance explained. Therefore, the final multiple linear regression analysis aimed to reflect a reduced set of predictors with minimal redundancy and stable parameter estimates. The results revealed that push-ups were a positive predictor of standing broad jump [*R*^2^ = 0.36, adjusted *R*^2^ = 0.36, *F* (1, 162) = 92.63, *p* < 0.001] (Table 3). This indicated that push-ups explained 36.0% of the variance in the standing broad jump.

**Table 3:** Time 1 predictor.

Source	*B*	SE *B*	95% CI		β	*t*	*p*	*r* ^2^	Adjusted *r*^2^
			LL	UL					
Fixed effects									
Intercept: standing broad jump	39.74	1.13	37.51	41.97		35.15	<0.001	0.36	0.36
Push-up	0.62	0.06	0.49	0.74	0.60	9.63	<0.001		

*N* = 164; CI, confidence interval; LL, lower limit; UL, upper limit.

Table 4 presents the means, standard deviations, and Pearson product correlations for the Time 2 variables. The Time 2 variables parallel the Time 1 variables, with positive, moderate, and statistically significant correlations among the following MusS assessments: standing broad jump and average single-leg three-hop [*r* (163) = 0.84, *p* < 0.01]; standing broad jump and push-up [*r* (163) = 0.62, *p* < 0.01]; and average single-leg three-hop and push-up [*r* (163) = 0.60, *p* < 0.01]. Resilience, however, was not moderately or strongly correlated to or statistically significant with any MusS or NC assessment.

**Table 4:** Relationship among MusS, NC, and resilience at Time 2.

Variable	*M*	SD	1	2	3	4	5
1. Average grip strength	36.71	8.90					
2. Push-up	12.99	9.82	0.20* [0.04, 0.34]				
3. Average single-leg three-hop	109.14	30.75	0.44** [0.30, 0.55]	0.60** [0.49, 0.69]			
4. Standing broad jump	48.69	10.48	0.46** [0.33, 0.57]	0.62** [0.51, 0.70]	0.84** [0.79, 0.88]		
5. Side-step	28.24	6.57	0.20** [0.05, 0.35]	0.34** [0.20, 0.47]	0.44** [0.31, 0.56]	0.43** [0.30, 0.55]	
6. Resilience	71.18	10.04	−0.06 [−0.21, 0.09]	0.09 [−0.06, 0.24]	0.06 [−0.09, 0.21]	0.06 [−0.09, 0.22]	0.14 [−0.02, 0.28]

* *p* < .05.

** *p* < .01.

In parallel with the analysis for Time 1 data, an inspection of the Time 2 variables revealed that the single-leg three-hop and push-up were similarly correlated to and predictive of the standing broad jump [*R*^2^ = 0.73, adjusted *R*^2^ = 0.73, *F* (1, 162) = 11.25, *p* < 0.001]. Similar to Time 1, the diagnostic indices suggested that multicollinearity was not severe (variance inflation facto*r* = 0.67, eigenvalue = <2.75, and condition index = 9.90). However, as the single-leg three-hop had the strongest correlation value and in parallel with the Time 1 analysis, the single-leg three-hop was removed from the regression analysis. Meanwhile, average grip strength was not a contributor to the prediction of the standing broad jump. Multiple linear regression analysis revealed that push-ups were a positive predictor of the standing broad jump [*R*^2^ = 0.38, adjusted *R*^2^ = 0.38, *F* (1, 162) = 9.98, *p* < 0.001] (Table 5). This indicated that push-ups explained 37.7% of the variance in the standing broad jump.

**Table 5:** Time 2 predictor.

Source	*B*	SE *B*	95% CI		β	*t*	*p*	*r* ^2^	Adjusted *r*^2^
			LL	UL					
Fixed effects									
Intercept: standing broad jump	40.13	1.07	38.01	43.35		37.38	<0.001	0.38	0.38
Push-up	0.66	0.07	0.53	0.79	0.62	9.98	<0.001		

*N* = 164; CI, confidence interval; LL, lower limit; UL, upper limit.

## 4 Discussion

### 4.1 Key findings

From Times 1 to 2, the correlations among MusS, NC, and resilience variables remained relatively the same, with slightly stronger correlations present at Time 2, suggesting that certain performance tests are related and predictive of one another. At both time points, positive, moderate, and statistically significant correlations were found between the standing broad jump and the average single-leg three-hop, the standing broad jump and push-ups, and the average single-leg three-hop and push-ups. The standing broad jump is the most standardized and commonly used assessment of the MusS battery, which measures bilateral lower-body explosiveness and power (2, 36). In contrast, single-leg three-hop is not standardized and has varied protocols in the literature; it measures unilateral lower-body power and agility (2, 34). However, it seems plausible that performance on one assessment would correspond to performance on the other, given that both are lower-body MusS assessments. Meanwhile, push-ups measure bilateral upper-body strength endurance (2, 43). The positive relationships between standing broad jumps and push-ups, as well as between the average single-leg three-hop and push-ups, support the overall idea that MusS and muscular endurance may influence body power and agility. This connection between the upper and lower body may be due to neuromuscular adaptations in the musculature, leading to superior strength (44). These correlations highlight the interconnected nature of these exercises. However, further research is needed to fully understand the relationship between MusS assessments.

At Time 1, push-ups were predictive of the standing broad jump, with 36.0% variance explained. In addition, at Time 2, push-ups were predictive of the standing broad jump, with 37.7% of the variance explained. The amount of variance was minimal (1.7%), but the variance may suggest a slightly enhanced connection between the upper-and lower-body MusS. The ICC value for push-ups was moderate (0.78), but it may not be the most reliable and replicable assessment for analyzing predictive variables. If the push-up reliability were higher, it was possible that the variance explained across time points would be greater. Notably, the period between the Times 1 and 2 data collection was only 4 months. Longitudinal studies might demonstrate increased variable variance if there was more time for children to gain upper- and lower-body MusS. Although recent literature remains scarce, Castro-Piñero et al. (45) also reported significant predictive associations between lower-body (standing broad jump and vertical jump) and upper-body (basketball throws, push-ups, and isometric strength) MusS in children. When predicting the standing broad jump and vertical jump, push-ups explained the least amount of variance (54.2% and 55.5%, respectively) compared with basketball throws (85.1% and 84.3%, respectively) and isometric strength (69.4% and 68.0%, respectively) (45). Future research should consider assessing MusS, NC, and resilience in fourth-and fifth-grade children participating in other recess interventions to compare with previous literature.

The side-step test (NC) had no strong or moderate, significant, or meaningful relationships with any MusS or resilience assessment. In fourth and fifth graders, NC relationships may not have been present since their MusS and motor control have not yet developed due to valgus limb alignment and a developing vestibular system (23, 46). In addition, the side-step had the lowest ICC value of all physical assessments; this moderate reliability could have impacted the relationships between the assessments. Therefore, alternative NC assessments should be performed to determine whether there is a more appropriate, suitable, and sensitive tool that assesses NC ability and produces greater reliability. However, because there were positive, weak, and statistically significant relationships with all MusS assessments, further research should investigate NC relationships in a larger, more diverse population of children while maintaining at least a 6-month gap between data points or collecting data three times per school year (September, January, and May).

Similarly, resilience also had no strong or moderate significant relationships with any MusS or NC assessment, suggesting that there are no meaningful relationships among resilience, MusS, or NC. Previous studies have revealed positive associations between MusS exercises (push-ups and sit-ups) and resilience, as those who engage in more MusS exercises have greater resilience scores (47). Zhang et al. (47) suggested that these associations may be induced as physical activities increase physiological responses (heart rate, blood pressure, and respiration) that are beneficial to the hypothalamic–pituitary–adrenocortical (HPA) axis, thereby improving psychological health. Similarly, Neumann et al. (48) reported that muscular fitness was positively associated with stress resilience. Therefore, the current findings contradict those of the recent literature, necessitating additional research on the use of resilience assessments and the relationship among resilience, MusS, and NC. Alternative or complementary resilience assessments should be considered to determine whether the attenuated observed effects were due to the limited number of months between data points and/or the assessments themselves.

### 4.2 Limitations

Several limitations should be considered when interpreting the findings of this study. First, there is a narrow body of literature on these variable relationships. The standing broad jump, average grip strength, and push-ups are the most standardized assessments; therefore, the existing literature focuses on these three assessments. However, there is little or no literature assessing the relationships among the single-leg three-hop, side-step, and resilience assessments and other MusS assessments. As such, while the findings of this study may be valuable, they should be interpreted with caution, as there is limited existing literature serving as a conceptual foundation. Further research is needed to validate these results and explore the nuances of these relationships and variable predictions. This study also serves as an exploratory study with preliminary results that are not generalizable to larger populations or contexts. Thus, the application of these results is restricted due to the limited sample size and specific study conditions. In addition, this study does not measure key confounders, including but not limited to height, weight, body composition [body mass index (BMI) or bioelectrical impedance analysis (BIA)], maturation stage, and PA outside of school, which could contribute to possible MusS relationships. Finally, the time span between data collection points was narrow, potentially being insufficient to notice growth or true longitudinal changes in MusS, NC, and resilience performance and relationships in this age group.

### 4.3 Practical implications and future research

Understanding the potential connected nature among MusS, NC, and resilience in fourth- and fifth-grade children holds meaningful implications, as identifying these relationships can lay the foundation for physical education and wellness programs, inside and outside the classroom. It can focus on determining the best resilience assessment to decipher the connections among MusS, NC, and resilience through other standardized assessments. Integrating curriculum and interventions based on these findings or similar findings could promote more holistic approaches to children's development that foster academic success with long-term physical and mental wellbeing. Finally, this study provides baseline data for educators and school professionals to identify children who may be at risk of poor physical or psychological outcomes.

Future research is needed to fully understand and validate the relationships among MusS, NC, and resilience in fourth- and fifth-grade children. Examining these preliminary findings in longitudinal studies would provide a broader understanding of the trajectories of these relationships. Extending the observation time or increasing the collection time point intervals would provide a sufficient duration to detect meaningful changes or trends in children's MusS, NC, and resilience. In addition, to make these findings more generalizable, the factors of age, sex, maturation, socioeconomic status, and environmental factors should be examined to assess the magnitude of their influence on these relationships. Furthermore, several studies have examined MusS with body mass index (BMI), leading to robust findings; however, BMI has not been shown to be as effective in determining body fat percentages in children. Therefore, the use of bioelectrical impedance analysis (BIA) in the field to assess its relationship with MusS, NC, and resilience variables may expand the scope of the data for a broader analytical perspective. Relatedly, the side-step and resilience assessments warrant continued validation efforts; thus, the use of alternative assessments that are more sensitive to this sample would provide a larger body of literature on these relationships. Finally, applying this research in diverse environmental, sports, socioeconomic, and educational settings would ensure that the findings are applicable to a variety of populations.

## 5 Conclusions

This study aimed to assess the relationships and variable predictions of MusS, NC, and resilience assessments in fourth- and fifth-grade children at two time points during one school year to measure the effectiveness of recess intervention. The results reveal moderate, positive, and significant correlations between the upper- and lower-body MusS assessments, with minimal predictive abilities and variance explained. Meanwhile, NC and resilience had no meaningful relationship with the other assessments. This exploratory study adds to the limited data available on MusS, NC, and resilience assessment relationships in children who participate in recess intervention. Future studies are needed to validate or contradict these findings and to continue examining the possible physical and psychological relationships in children.

## Data Availability

The raw data supporting the conclusions of this article will be made available by the authors, without undue reservation.
